# An efficient piecewise linear model for predicting activity of caspase-3 inhibitors

**DOI:** 10.1186/2008-2231-20-31

**Published:** 2012-09-10

**Authors:** Loghman Firoozpour, Khadijeh Sadatnezhad, Sholeh Dehghani, Eslam Pourbasheer, Alireza Foroumadi, Abbas Shafiee, Massoud Amanlou

**Affiliations:** 1 Department of Medicinal Chemistry, Faculty of Pharmacy and Pharmaceutical Sciences Research Center, Tehran University of Medical Sciences, Tehran, Iran; 2 Computer & IT Engineering, AmirKabir University of Technology, Tehran, Iran; 3 Drug Design & Development Research Center, Tehran University of Medical Sciences, Tehran, Iran

**Keywords:** Alzheimer’s disease, QSAR, Apoptosis, XCSF, ANN, Caspase-3

## Abstract

**Background and purpose of the study:**

Multimodal distribution of descriptors makes it more difficult to fit a single global model to model the entire data set in quantitative structure activity relationship (QSAR) studies.

**Methods:**

The linear (Multiple linear regression; MLR), non-linear (Artificial neural network; ANN), and an approach based on “Extended Classifier System in Function approximation” (XCSF) were applied herein to model the biological activity of 658 caspase-3 inhibitors.

**Results:**

Various kinds of molecular descriptors were calculated to represent the molecular structures of the compounds. The original data set was partitioned into the training and test sets by the K-means classification method. Prediction error on the test data set indicated that the XCSF as a local model estimates caspase-3 inhibition activity, better than the global models such as MLR and ANN. The atom-centered fragment type CR_2_X_2_, electronegativity, polarizability, and atomic radius and also the lipophilicity of the molecule, were the main independent factors contributing to the caspase-3 inhibition activity.

**Conclusions:**

The results of this study may be exploited for further design of novel caspase-3 inhibitors.

## Introduction

There are some challenges such as new disease and drug resistance which our society is faced with. On the other hand, drug discovery is a costly and time consuming process. In this context, there is a great demand for predictive models to design new drugs with improved properties and diminished side effects [1]. Furthermore, there is also a demand for new methods that replace and reduce the use of laboratory animals [2]. These methods should be used in the design and evaluation of experimental tests and in the selection of appropriate test compounds for validation studies [3].

In order to reduce the time and cost during the drug development process, quantitative structure activity relationship (QSAR) is a robust scientific method which can help scientists to predict the activity and side effects of new compounds [4,5]. Distribution of training data affects on the model that is suitable for it, so searching to find a convenient method is an essential part of QSAR model evaluation to predict the biological activity for new targets and new compounds.

Herein, we described a linear (Multiple linear regression, MLR) and one non-linear (Artificial neural network, ANN) as global models and Extended Classifier System in Function approximation (XCSF) as a local model to develop a convenient model for predicting caspase-3 inhibition activity [6]. Caspases are cysteine-aspartic specific proteases involved in the signaling cascades of programmed cell death. Caspase-3 is a key executioner member of the caspase family which propagates death signals from intrinsic and extrinsic stimuli to downstream targets. Inappropriate control of caspases (especially caspase-3) as apoptosis machinery has been implicated in many diseases, including neurodegenerative disorders, cancer, and autoimmune diseases [7,8]. In this study, we tried to develop and choose a robust model to predict caspase-3 inhibition activity as a scientific method to facilitate the discovery of anti-neurodegenerative agents.

## Methods

### Biological data and descriptors generation

Structures of caspase-3 inhibitors and corresponding biological activities were downloaded from Binding database in MDL, in SD file format, which were then extracted and converted to corresponding Mol format files [9]. In total 658 structures with experimentally tested IC_50_ were used as input data in this study. After geometry optimization of the molecular structures, the Dragon software (version 2.1) was used to calculate descriptors. In total 1481 descriptors (variables) were calculated by this procedure.

### Feature selection and linear regression

In order to reduce the number of descriptors, a feature selection algorithm was carried out in four steps. In the first step, variables with more than 80% constant values were removed. For the second step, variables with less than 0.7 correlations with biological activity (caspase-3 inhibition activity) were omitted.

In the third step, descriptors which showed 0.7 cross correlation with each other were removed from variables. After these three steps, 99 variables remained in the data set, but they were more than what was required for QSAR models. For solving this problem, multivariate linear regression was implemented and stepwise selection method was used as the fourth step. 24 descriptors remained after the fourth step and were used for the linear model (24 remained descriptors are shown in Table 1). Mean square error of linear regression model on the training set is 0.262.

**Table 1:** Selected variables after 4 feature selection steps

**No**	**Variable code**	**Variable category**	**Variable dimension**
1	N-072	Atom centered fragments	1D
2	C-012	Atom centered fragments	1D
3	O-060	Atom centered fragments	1D
4	nCO	functional groups	1D
5	nCrR2	functional groups	1D
6	MLOGP	properties	1D
7	MATS4v	2D autocorrelations	2D
8	GATS8p	2D autocorrelations	2D
9	GATS6e	2D autocorrelations	2D
10	ATS5e	2D autocorrelations	2D
11	MATS7e	2D autocorrelations	2D
12	SIC0	Topological descriptors	2D
13	piPC10	Topological descriptors	2D
14	PCD	Topological descriptors	2D
15	PJI2	Topological descriptors	2D
16	Mor31m	3D-MoRSE descriptors	3D
17	Mor17e	3D-MoRSE descriptors	3D
18	Mor12p	3D-MoRSE descriptors	3D
19	HOMA	aromaticity indices	3D
20	HATS1u	Getway descriptors	3D
21	R5e+	Getway descriptors	3D
22	R4e	Getway descriptors	3D
23	H8e	Getway descriptors	3D
24	RDF070m	RDF descriptors	3D

1D, 2D, 3D and others (charge descriptors and molecular properties) correspond to the type of molecular descriptor selected [10].

For the non-linear models the 24 selected variables, which have been selected by linear regression, are too many, so in the next step principal component analysis (PCA) on 24 selected variables was applied. After this step, seven variables were selected (See Table 2).

**Table 2:** Selected variables after principal component analysis (Factor analysis)

**No**	**Variable code**	**Variable category**	**Variable dimension**
1	C-012	Atom centered fragments	1D
2	MLOGP	properties	1D
3	GATS6e	2D autocorrelations	2D
4	PJI2	Topological descriptors	2D
5	Mor12p	3D-MoRSE descriptors	3D
6	R4e	Getway descriptors	3D
7	RDF070m	RDF descriptors	3D

As shown in Table 2, after PCA, one descriptor was selected among each category of descriptors and some categories were eliminated completely. Among selected descriptors, 3D ones have more contribution rather than 1D and 2D ones, which confirms the role of spatial restrictions of caspase-3 receptor to pick up its inhibitors.

### Extended classifier system in function approximation

XCSF is a piecewise linear function approximation system which approximates function values in the continuous space [1,11]. There are some differences between XCSF and XCS representation of conditions, classifier prediction mechanism, update process are some essential difference points between XCSF and XCS [12].

In XCSF, each classifier consists of a condition, an action, and some other parameters. The condition covers input states that the classifier matches with them. The action specifies the action for which the payoff is predicted and it is executed on environment. XCSF as a pure function approximator has a dummy action which has no actual effect on the environment. The main XCSF parameters are: the weight vector$,\vec{w}\text{,}$ that is used to compute the classifier prediction of input state; the prediction error,ε, which estimates the error affecting classifier prediction; the numerosity, *num*, used to determine number of different copies of the same classifier. The weight vector$,\vec{w}\text{,}$ which has *n* + 1 components in *n* dimensional state space (Each *w*_*i*_ is corresponding to a special feature of input space and *w*_0_ is corresponding to a constant input, *x*_0_, which is set as a parameter of XCSF).

Performance component of XCSF works as XCS. At each time step XCSF builds a *match set,* [M], containing the classifiers in the classifier list or population, [P], whose condition matches with current input state; if [M] contains less than *θ*_*mna*_ classifiers, *covering* process occurs as in XCSI^12^; making a classifier that matches with current state and inserting it to [M]. In the covering process, the weight vectors of classifiers are initialized with zero values; all the other parameters are initialized as in XCS [13]. In XCSF as a pure function approximator, prediction is computed by the fitness-weighted average of all matching classifiers:

(1) $$P\left(s_{t}\right)=\frac{\sum_{cl\in \left[M\right]cl.F*cl.p\left(s_{t}\right)}}{\sum_{cl\left[M\right]cl.F}}$$

Where *s*_*t*_ is current sensory input, *cl* is a classifier, [M] represents match set, *cl*.*F* is the fitness of *cl* classifier, and *cl*.*p*(*s*_*t*_) is the prediction of *cl* in state *s*_*t*_ which is computed as:

(2) $$cl.p\left(s_{t}\right)=cl.w_{0}*x_{0}+\sum_{i>0}cl.w_{i}*s_{t}\left(i\right)$$

Where *s*_*t*_ is current sensory input, *cl*.*w*_*i*_ is the weight *w*_*i*_ of *cl*, and *i* is related to *i*’th dimension of input state. XCSF uses the reward value *P* (actual function value for current input) to update the parameters of classifiers in *match set*. The weight vector *w* of the classifiers in [M] are updated using a *modified delta rule:*

(3) $$\Delta w_{i}=\frac{\eta }{{\left|s_{t}\left(i\right)\right|}^{2}}*\left(P-cl.p\left(s_{t}\right)\right)s_{t}\left(i\right)$$

Where *η* is the correction rate, |*s*_*t*_(*i*)|^2^ is the norm input vector *s*_*t*_[1]. The weights of classifier *cl* are updated using *Δw*_*i*_ values as:

(4) $$cl.w_{i}=cl.w_{i}+\Delta w_{i}$$

Then the prediction error *ε *is updated as:

(5) $$cl.\varepsilon =cl.\varepsilon +\beta \left(\left|P-cl.p\left(s_{t}\right)\right|-cl.\varepsilon \right)$$

Where *cl*.*ε *is the prediction error of classifier *cl*, *β* is learning rate, and *P* is the reward value. Classifier fitness is updated similar to XCS.

The genetic algorithm in XCSF works as in XCSI^1^. Genetic algorithm (GA) is applied to improve the rule set of XCSF by generating new classifiers which contribute to existing knowledge and removing classifiers which do not offer any improved contributions. In function approximation, the genetic algorithm (GA) is applied to the classifiers of match set [M]. Firing of GA component is directly depending on *θ*_*ga*_. Two classifiers are selected as parents with the probability proportional to their fitness. Crossover performed with a fixed probability *p*_*c*_ on copies of individuals and with probability *p*_*m*_ mutation changes their allele. Before inserting off springs to the population set, in order to keep a fixed population size, two classifiers may be deleted. For a sufficient experienced and accurate classifier deletion probability is proportional to its set size and fitness. Hence, if an experienced classifier has lower fitness rather than average fitness of classifiers in population set, its deletion probability is increased [11,13]. So, generation of maximally general conditions that efficiently cover the feature space is performed by GA progress.

### Artificial neural network

To examine the ability of 7 selected features in predicting activity values of inhibitors, selected variables using feature selection filters are fed into input layer of ANN. A three-layer neural network with 7-X-1 structure is used in this study. ANN parameters were optimized according to trial-and-error procedure. Data set were divided to training, validation, and test subsets. Validation set results directed us to find optimal setting for ANN. To access the performance of fully- trained model, test samples are evaluated and after evaluating the final model by using the test set, the model parameters should not tune further.

## Results and discussion

The proper selection of a training set is one of the most basic operations in quantitative structure activity relationship studies. Small, relevant, and homogeneous data sets have and continue to be the workhorse for structure-activity predictions when the activity for a new analogue is needed for a particular chemical series. For large data sets, however, the selection of a training set is critical since compounds of diverse chemical structure are contained within the chemical space of the database.

To remove the dependency between the training and testing samples, 10-fold cross validation is performed [14]. The original samples are randomly partitioned into *10 s*ubsets. Of the *10* subsamples, a single subsample is retained for testing the model, and the remaining *9* subsamples are used in the training process. The cross-validation process is repeated *10* times, so each of the *10* subsamples used exactly once as the validation data. All observations are used for both training and validation sets, and each observation is used for validation exactly once.

In this study, in a preprocessing phase, training data are partitioned into multiple clusters (by the *K*-means algorithm) [14]. Optimal number of clusters is determined by the Silhouette method [15]. Average distance between each training sample and center of its corresponding cluster is considered as stretch variation in condition creation. The achieved results using XCSF classifier is computed by averaging among 20 experiments. The XCSF parameters are set as *N* = 656, *maxPopSize* = 30, *functionSize* = 7, *coverConditionRange* in this problem is approximately 0.17, *realConditionType* is *hyperEllipsoidalCondition, ellipsoidalConditionType* is *axis-parallel ellipsoid, type of prediction* is *linear prediction with RLS update* and other parameters are set according to reference [16].

**Figure 1:**
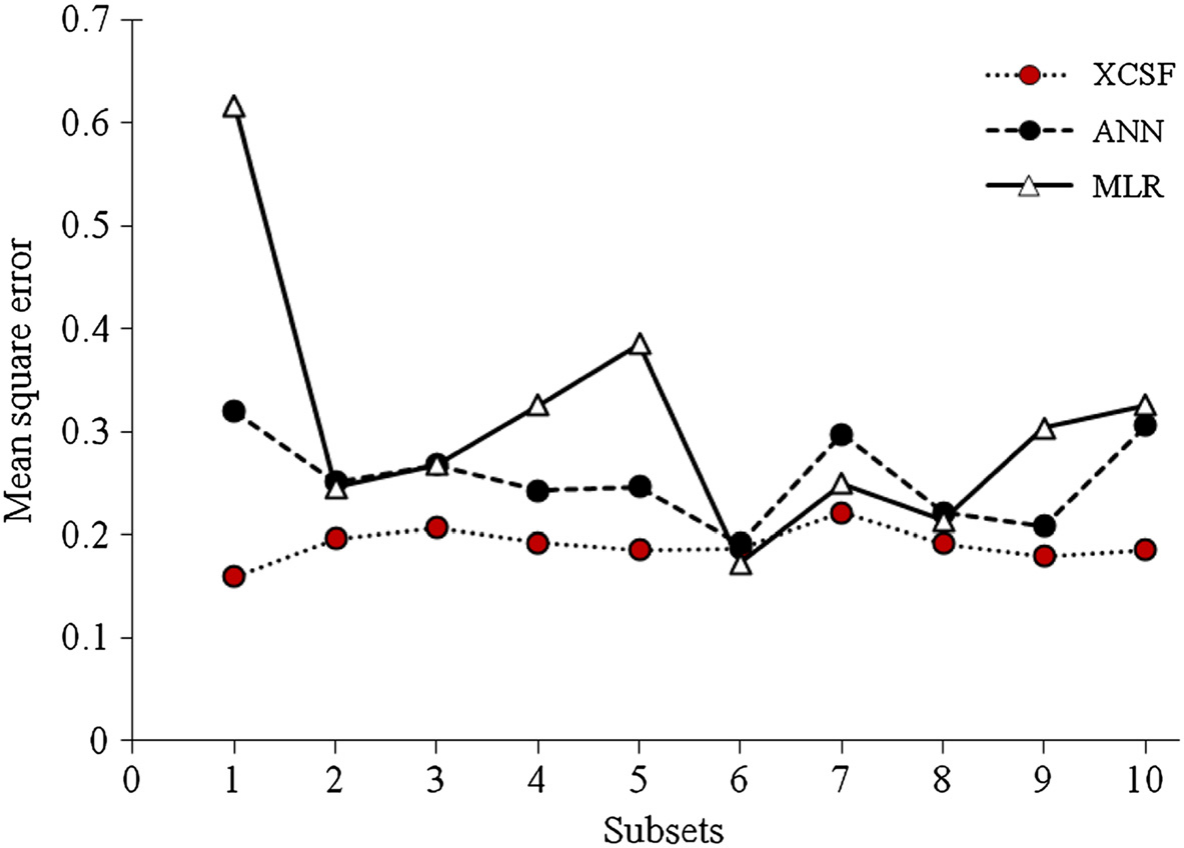
Plot of the mean square errors of prediction versus the 10 subsets by XCSF, ANN and MLR methods.

Mean square error (MSE) of each method has been calculated for all 10 subsets (See Table. 3). The plot of the mean square errors versus the 10 subsets is shown in Figure 1. As depicted, the XCSF models are superior to both of the MLR and ANN models. Mean of these errors has been used to calculate *P*-values (See Table. 4). Table 4 shows that the differences between XCSF and ANN and also between XCSF and MLR are significant. According to Tables 3 and 4, the XCSF model is an appropriate model with accepted differences from the with MLR and ANN models.

**Table 3:** Mean square errors of linear and non-linear models

**Subsets**	**Models**		
	**MLR**	**XCSF**	**ANN**
Subset-1	0.6163	0.16	0.3202
Subset-2	0.246	0.196	0.2509
Subset-3	0.2678	0.207	0.2678
Subset-4	0.3259	0.192	0.2428
Subset-5	0.3857	0.186	0.2464
Subset-6	0.1724	0.187	0.1918
Subset-7	0.25	0.221	0.2979
Subset-8	0.2135	0.192	0.2216
Subset-9	0.304	0.18	0.2085
Subset-10	0.326	0.185	0.3063
Mean	0.3108	0.1907	0.2554
Standard deviation	0.1237	0.0163	0.0427

**Table 4:** ***P*-values for applied models**

**Manner**	***P*-value**	**α**	**State**
XCSF & ANN	0.002	0.05	Δ
XCSF & MLR	0.02	0.05	Δ
ANN & MLR	0.124	0.05	-

### Descriptors description

Besides demonstrating statistical significance, QSAR models should also provide useful chemical insights for drug design. For this reason, an acceptable interpretation of the QSAR results is provided below. By interpreting the descriptors contained in the model, it is possible to gain some insight into the factors which are related to the caspase-3 inhibition activity.

C-012 is the first descriptor, appearing in the model. It is one of the atom-centered fragment descriptors that describes each atom by its own atom type, the bond types and atom types of its first neighbors. The C-012 descriptor displays CR_2_X_2_. This atom-centered fragment descriptor can be defined as a central carbon atom (C) that has two carbon neighbors (R_2_) and two heteroatom neighbors (X_2_).

The second descriptor is MLOGP which is one of the molecular properties descriptors. The molecular descriptor MLOGP refers to the Moriguchi log of the octanol/water partition coefficient of the molecule and is considered as a measure of lipophilicity of a molecule.

The next descriptor is GATS6e (Geary autocorrelation - lag 6 / weighted by atomic Sanderson electronegativities), which is one of the 2D autocorrelations descriptors. In this descriptor the Geary coefficient is a distance-type function, that function is any physico-chemical property calculated for each atom of the molecule, such as atomic mass, polarizability, etc. Therefore, the molecule atoms represent the set of discrete points in space and the atomic property the function evaluated at those points. The physico-chemical property in this case is atomic Sanderson electronegativities.

The 2D Petitjean shape index (PJI2), is one of the topological descriptors. This descriptor also called graph-theoretical shape coefficient, is proposed to describe the topological anisometric. This molecular shape descriptor describes the degree of deviation from a perfect cyclic topology.

Mor12p is the next descriptor, appearing in the model. It is one of the 3D-MoRSE descriptors. 3D MoRSE descriptors (3D Molecule Representation of Structures based on Electron diffraction) are derived from Infrared spectra simulation using a generalized scattering function. This descriptor was proposed as signal 12 / weighted by atomic polarizability which relates to polarizability of the molecules.

The sixth descriptor of the model was the R autocorrelation of lag 4 / weighted by atomic Sanderson electronegativities (R4e). It belongs to the GETAWAY descriptors. This descriptor is related to the electronegativity, the size and the location of the atom in the molecule. The greater the electronegativity, atomic radius and the distance between the atom and the center of the molecule are, the greater the descriptor value is.

The final descriptor is RDF070m (Radial Distribution Function - 7.0 / weighted by atomic masses), which is one of the radial distribution function (RDF) descriptors. RDF in this form meets all the requirements for the 3D structure descriptors. It is independent of the atom number (i. e. the size of a molecule), and is unique regarding the three-dimensional arrangement of the atoms and is also invariant against the translation and rotation of the entire molecule. Additionally, the RDF descriptors can be restricted to specific atom types or distance ranges to represent specific information in a certain three-dimensional structure space.

In summary, it is concluded that; the atom centered fragment type CR_2_X_2_, electronegativity, polarizability, size of atoms and the lipophilicity of the molecule play a main role in the caspase-3 inhibition activity of the studied compounds.

## Conclusion

In this study we compare three global and local QSAR modeling techniques in caspase-3 activity prediction. K-means and Silhouette method show that data set is distributed in different locality of feature space. Linear regression as a global method without using the information of different clusters that are scattered in the data set tunes a single hyper plane to predict the activity function. Applying global nonlinear methods such as neural networks can be more useful than global linear methods. Neural networks try to match its prediction models to entire data set precisely so that may cause over training error. Multimodal distribution of features generated from caspace-3 data set causes to apply a local model which tunes a modeling system on each cluster of data set. XCSF as a piecewise linear system in function approximation problems is used for prediction of activity value corresponding to each sample. As expected, results show that global nonlinear method predicts activities more accurate than global linear regression method and a local model that regards the locality information is the most accurate. Results are presented for caspase-3 data indicating that XCSF as a local modeling strategy is preferred over a global strategies such as linear regression and ANN.

## Competing interest

There are no other conflicts of interest related to this publication.

## Authors’ contribution

All authors contributed to the concept and design, making and analysis of models, drafting, revising and final approval. MA is responsible for the study registration. LF and MA for conception and design, LF, KhS, ShD and EP for provision of study material, LF, KhS, ShD and EP for collection and/or assembly of data, data analysis, interpretation and manuscript writing and AF, ASh and MA for financial and administrative support. All authors read and approved the final manuscript.

## References

[B1] Lokwani D Bhandari S Pujari R Shastri P Xshelke G Pawar V Use of Quantitative Structure–Activity Relationship (QSAR) and ADMET prediction studies as screening methods for design of benzyl urea derivatives for anti-cancer activity J Enzym Inhib Med Chem 2011 26 319 331 10.3109/14756366.2010.506437 20846089

[B2] Ravichandran V Mourya VK Agrawal RK Prediction of HIV-1 protease inhibitory activity of 4-hydroxy-5,6-dihydropyran-2-ones: QSAR study J Enzym Inhib Med Chem 2011 26 288 294 10.3109/14756366.2010.496364 20735159

[B3] Sprous DG Fingerprint-based clustering applied to define a QSAR model use radius J Mol Graphics Model 2008 27 225 232 10.1016/j.jmgm.2008.04.009 18556228

[B4] Shahlaei M Fassihi A Saghaei L Arkan E Pourhossein A A QSAR study of some cyclobutenediones as CCR1 antagonists by artificial neural networks based on principal component analysis Daru 2011 19 376 384 22615684 PMC3304395

[B5] Foroumadi A Sakhteman A Sharifzadeh Z Mohammadhosseini N Hemmatinejad B Moshafi MH Vosooghi M Amini M Shafiee A Synthesis, antituberculosis activity and QSAR study of some novel 2-(nitroaryl)-5-(nitrobenzylsulfinyl and sulfonyl)-1,3,4-thiadiazole derivatives Daru 2007 15 218 226

[B6] Wilson SW Classifiers that approximate functions J Nat Computing 2002 1 211 234

[B7] Fan TJ Han LH Cong RS Liang J Caspase family proteases and apoptosis Acta Biochimica Biophysica Sinica 2005 37 719 727 10.1111/j.1745-7270.2005.00108.x 16270150

[B8] Sharma S Ravichandran V Jain PK Mourya VK Agrawal RK Prediction of Caspase-3 inhibitory activity of 1,3-dioxo-4-methyl-2,3-dihydro-1 h-pyrrolo[3,4-c] quinolines: QSAR study J Enzym Inhib Med Chem 2008 23 424 431 10.1080/14756360701652476 18569350

[B9] Liu T Lin Y Wen X Jorissen RN Gilson MK Binding DB A web-accessible database of experimentally determined protein–ligand binding affinities Nucleic Acids Res 2007 35 198 201 10.1093/nar/gkl999 PMC1751547 17145705

[B10] Todeschini R Consonni V Handbook of Molecular Descriptors 2000 Weinheim: Wiley-VCH

[B11] Lanzi PL Loiacono D Wilson SW Goldberg DE Extending XCSF Beyond Linear Approximation 2005 New York: ACM New York 1827 1834

[B12] Wilson SW Classifier fitness based on accuracy Evol Comput 1995 3 149 175 10.1162/evco.1995.3.2.149

[B13] Butz MV Wilson SW An algorithmic description of XCS J Soft Computing 2002 6 144 153 10.1007/s005000100111

[B14] Devijver PA Kittler J Pattern Recognition: A Statistical Approach 1982 London, GB: Prentice-Hall

[B15] Rousseeuw PJ Silhouettes: a graphical aid to the interpretation and validation of cluster analysis Comput Appl Math 1987 20 53 65

[B16] Butz MV Pedersen GKM Stalph PO Learning sensorimotor control structures with XCSF: redundancy exploitation and dynamic control 2009 New York: ACM New York 1171 1178

